# TMEM184B modulates endolysosomal acidification via the vesicular proton pump

**DOI:** 10.1242/jcs.263908

**Published:** 2025-08-08

**Authors:** Elizabeth B. Wright, Erik G. Larsen, Marco Padilla-Rodriguez, Paul R. Langlais, Martha R. C. Bhattacharya

**Affiliations:** ^1^ University of Arizona, Department of Neuroscience, 1040 E 4th Street, Tucson, AZ 85721, USA; ^2^ Graduate Interdisciplinary Program in Neuroscience, University of Arizona, Tucson, AZ 85721, USA; ^3^ University of Arizona Cancer Center, 1515 N Campbell Ave, Tucson, AZ 85724, USA; ^4^ University of Arizona Department of Medicine, 1501 N. Campbell Ave, Tucson, AZ 85724, USA

**Keywords:** Endosome, Lysosome, Neuron, V-ATPase, TMEM184B, Acidification

## Abstract

Disruption of endolysosomal acidification causes toxic protein accumulation and neuronal dysfunction linked to neurodevelopmental and neurodegenerative disorders. However, the molecular mechanisms regulating neuronal endolysosomal pH remain unclear. Transmembrane protein 184B (TMEM184B) is a conserved seven-pass transmembrane protein that is essential for synaptic function, and its sequence disruption is associated with neurodevelopmental disorders. Here, we identify TMEM184B as a key regulator of endolysosomal acidification. TMEM184B localizes to early and late endosomes, and proteomic analysis confirms that TMEM184B interacts with endosomal proteins, including the vacuolar ATPase (V-ATPase), a multi-subunit proton pump crucial for lumenal acidification. *Tmem184b-*mutant mouse cortical neurons have reduced endolysosomal acidification compared to wild-type neurons. We find reductions in V-ATPase complex assembly in *Tmem184b*-mutant mouse brains, suggesting that TMEM184B facilitates endosomal flux by promoting V-ATPase activity. These findings establish TMEM184B as a regulator of neuronal endolysosomal acidification and provide mechanistic insight into its role in TMEM184B-associated nervous system disorders.

## Teaser

**Summary:** TMEM184B is an endolysosomal transmembrane protein that interacts with subunits of the V-ATPase proton pump and contributes to proper lumenal acidification in neurons.

## INTRODUCTION

Decreases in endolysosomal acidification contribute to cellular dysfunction in many neurological diseases. Cellular models of lysosomal storage disorders (LSDs), Alzheimer's disease (AD), Nieman–Pick type C disease (NPCD), and amyotrophic lateral sclerosis (ALS) all exhibit endolysosomal deacidification that causes the accumulation of toxic proteins (Arbo et al., 2020; Hu et al., 2015; Im et al., 2023; Lee et al., 2022; Lloyd-Evans et al., 2008; Lo and Zeng, 2023; Root et al., 2021; Vivas et al., 2019) Enlarged endosomal compartments or accumulation of autophagic vesicles in neurons occurs frequently in neurological diseases, implying that cargo delivery or degradation is impaired (Cataldo et al., 1996, 2000; Nixon and Rubinsztein, 2024; Root et al., 2021). How endolysosomal deacidification initially occurs, and the mechanistic link between deacidification and disease pathogenesis, is not fully understood.

Endosomal trafficking relies on lumenal acidification mediated by the vesicular proton pump (V-ATPase). The V-ATPase consists of two complexes – the membrane-embedded V_0_ complex and the cytoplasmic V_1_ complex (Cotter et al., 2015; Forgac, 2007; Toei et al., 2010). When assembled, the V_1_ complex hydrolyzes ATP, driving the translocation of protons through the V0a subunits into the lumen. This proton transport gradually reduces the lumenal pH, facilitating compartment progression through the endolysosomal pathway. The dynamic disassembly and assembly of the V-ATPase is tightly regulated by various factors, including amino acids, cholesterol and intracellular signaling pathways (Hurtado-Lorenzo et al., 2006; Zoncu et al., 2011; Kane, 2012; Colacurcio and Nixon, 2016; Ratto et al., 2022) Resident transmembrane proteins, such as the arginine transporter SLC38A9, can also modulate V-ATPase activity by sensing charged molecules, balancing proton accumulation in the lumen (Chadwick et al., 2021; Wyant et al., 2017).

Transmembrane protein 184B (TMEM184B) is an evolutionarily conserved seven-pass transmembrane protein broadly expressed throughout the nervous system, with predominant expression in neurons (Bhattacharya et al., 2016; Larsen et al., 2022; Wright et al., 2023). Mice with a gene-trap disruption in the TMEM184B genomic locus (resulting in <5% mRNA expression compared to wild type; hereafter called *Tmem184B*-mutant mice) have swollen presynaptic terminals and disrupted synaptic gene expression, while flies with disruptions in the ortholog *Tmep* have extra terminal branching, impaired synaptic transmission and concomitant disruptions in behavior (Bhattacharya et al., 2016; Cho et al., 2022). Electron microscopic analysis shows multilamellar inclusions within mutant presynaptic terminals (Bhattacharya et al., 2016), suggesting a possible disruption in endolysosomal maturation or transport. Finally, human variants of TMEM184B have been linked to a neurodevelopmental syndrome characterized by developmental delay, structural brain defects including corpus callosum hypoplasia and microcephaly, and seizures. Cells expressing pathogenic variants of TMEM184B show disrupted cellular metabolism and enhanced nuclear localization of the stress-responsive transcription factor EB (TFEB) (Chapman et al., 2024 preprint), suggesting that TMEM184B might promote metabolism linked to endolysosomal flux. However, the mechanism by which TMEM184B supports neuronal structure and function through endolysosomal flux remains unclear.

In this study, we investigated the functional role of TMEM184B in the endolysosomal system. We found that TMEM184B co-immunoprecipitated with proteins known to regulate intracellular transport and endolysosomal trafficking, including the V-ATPase. Neurons from *Tmem184b*-mutant mice had an elevated endolysosomal pH compared to wild-type controls, highlighting a role in endolysosomal function. We measured disruptions to V-ATPase subunit assembly in mutant tissue, which suggested that a reduction of V-ATPase activity occurs in the absence of TMEM184B. These findings identify a mechanistic basis by which TMEM184B regulates neuronal endolysosomal acidification to ensure synaptic structure and function. Our data also suggest an explanation for how TMEM184B functional alteration disrupts neural development.

## RESULTS AND DISCUSSION

### TMEM184B co-immunoprecipitates with multiple proteins involved in endosomal trafficking and autophagy

**Fig. 1.**
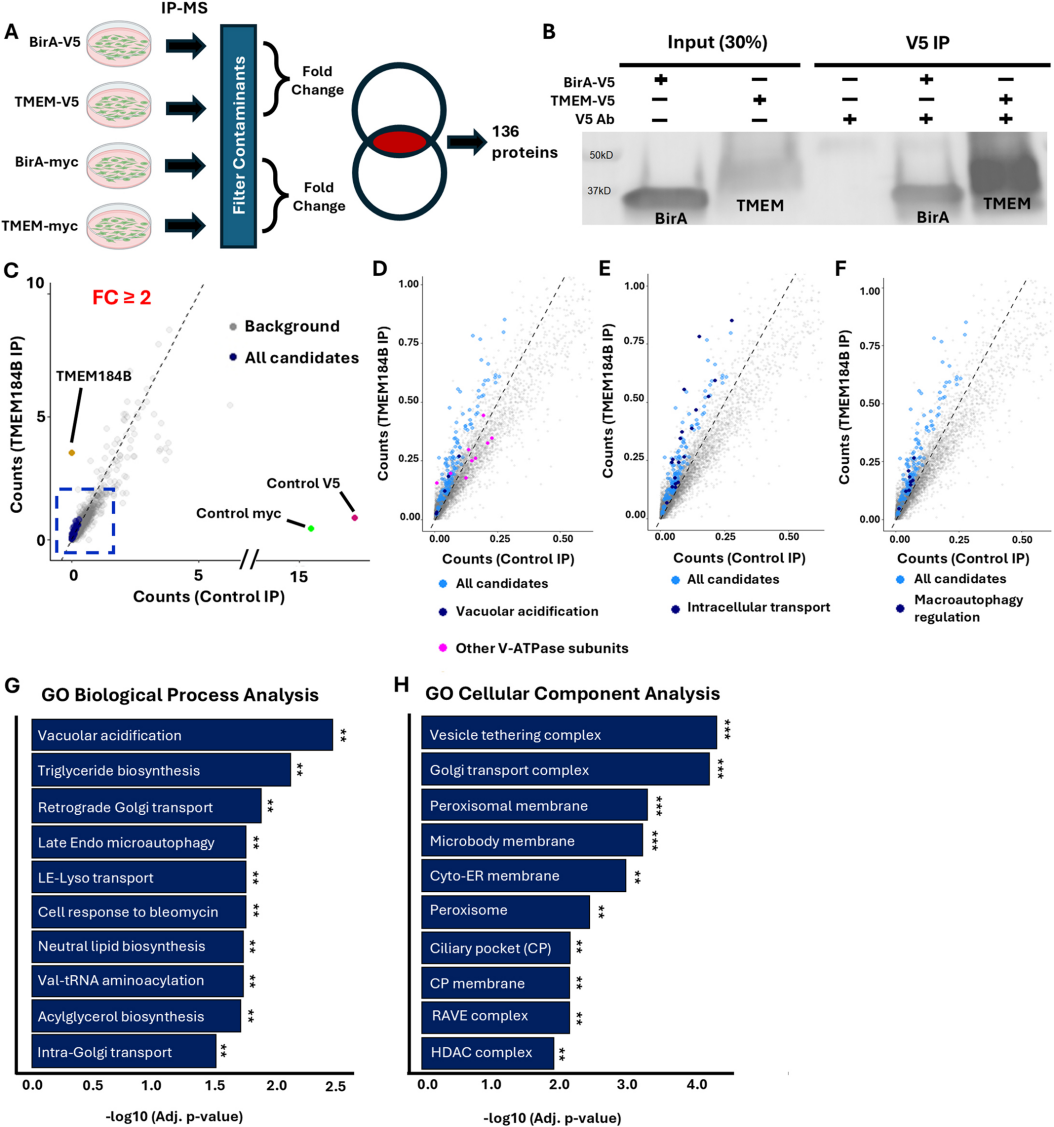
**TMEM184B co-immunoprecipitates with multiple proteins involved in endosomal trafficking and autophagy.** (A) Workflow of IP-MS analysis. (B) Western blot showing successful pulldown of TMEM184B (V5 tag). Blot representative of two independent biological replicates. (C) Average spectral counts of TMEM184B versus control (Myc or V5) immunoprecipitation from HEK293T cells. Data are from four independent IP-MS experiments using two different affinity tags with two biological replicates each. Enriched interactors with TMEM184B are shown in blue; background proteins in gray; TMEM184B in gold. Dashed box highlights the 136 enriched proteins. All points above and left of the dotted line have fold change >2. (D–F) Cropped scatterplots from C showing enriched proteins within functional categories. Light blue represents all proteins; dark blue, proteins in the functional category. In D, proteins associated with vacuolar acidification are shown in pink. (G,H) Top 10 gene ontology (GO) terms for biological processes (G) and cellular components (H) for the 136 significant IP-MS hits, sorted by fold enrichment and adjusted *P*-value. ***P<0.001; **P<0.01 (Fisher's exact test).

To clarify the role of TMEM184B in cellular processes that could contribute to neuronal maintenance (Bhattacharya et al., 2016; Cho et al., 2022), we first sought to identify candidate interacting proteins within human cells. Because currently available antibodies show nonspecific binding in mutant tissue (M.R.C.B., data not shown) we could not cleanly immunoprecipitate endogenous TMEM184B from human or mouse tissue. We therefore performed immunoprecipitation (IP) of overexpressed, tagged TMEM184B with tandem mass spectrometry (MS/MS). We performed the IPs with two different antibodies against independent epitope tags (V5 and Myc), each with two biological replicates, to create multiple TMEM184B interactomes that act as cross references and improve identification of potential TMEM184B interaction partners (Fig. 1A,B) (Kruse et al., 2017). Negative control IPs used biological replicates of a bacterial protein (BirA) attached to V5 or Myc. After filtering out common contaminants (Mellacheruvu et al., 2013) and intersecting potential candidate interactors from both datasets, we identified 136 unique proteins as candidate interactors of TMEM184B (Fig. 1C–F; Table S1).

Interestingly, TMEM184B co-immunoprecipitated with two isoforms of the V-ATPase subunit V0a (a1 and a2) [fold change (FC)=3.38 and 3.18, respectively]. TMEM184B has not been identified before in association studies that defined the proteins contributing to the V-ATPase subcomplexes or their regulatory proteins (Merkulova et al., 2015). Therefore, to confirm a potential interaction between FLAG–TMEM184B–GFP and ATP6V0A1 (the V0a1 subunit), we performed co-IP assays (Fig. S1). We used V0a1 as bait and probed for FLAG-tagged proteins (GFP or TMEM184B), followed by the reverse IP using FLAG antibody followed by blotting for V0a1. This approach confirmed an association between V0a1 and TMEM184B. Regulators of V-ATPase assembly were also found among the interacting protein candidates, including the *Drosophila melanogaster* X chromosomal gene-like proteins (DMXL1 and DMXL2), also known as Rabconnectin-3 (FC=4.05 and 4.44, respectively) (Eaton et al., 2024). Together, these data suggest that TMEM184B and the V-ATPase are associated in human cells.

Among hits in the mass spectrometry analysis, many proteins were involved in cellular trafficking or autophagy, suggesting a possible influence of TMEM184B in these dynamic processes (Table S1). Notably, proteins involved in regulating endosomal trafficking to the plasma membrane were enriched in TMEM184B IP samples, including the ADP-ribosylation factor guanine nucleotide exchange factor 2 (ARFGEF2, FC=4.72), the AAA-ATPase VPS4B (FC=3.25), coiled-coil domain containing 22 (CCDC22, FC=3.43) and EH domain containing protein family 3 (EHD3, FC=3.35) (Bar et al., 2013; Singla et al., 2019; Tseng et al., 2021; Zhu et al., 2022). Collectively, these proteins contribute to endosomal trafficking, each fulfilling a distinct function in vesicle trafficking, protein recycling, and the maintenance of cellular homeostasis. The autophagy-related proteins tuberous sclerosis complex 2 (TSC2, FC=2.55) and Activating molecule in BECN1-regulated autophagy protein 1 (AMBRA1, FC=2.433) were also present within TMEM184B immunoprecipitates. These proteins regulate autophagy through distinct mechanisms – TSC2 modulates mTORC1 and AMPK signaling pathways, and AMBRA1 directly participates in autophagosome formation (Albishi and Palli, 2023; Di Bartolomeo et al., 2010; Di Nardo et al., 2014; Li et al., 2022; Ng et al., 2011).

To identify enriched functional categories of TMEM184B-interacting protein candidates, we performed Gene Ontology (GO) analyses for biological processes and cellular components. Multiple categories indicated involvement in vacuolar acidification and intracellular transport (Fig. 1G). Cellular components enriched in TMEM184B interactors included vesicle tethering and transport complexes as well as the regulator of the H^+^-ATPase of vacuolar and endosomal membranes (RAVE) complex (Fig. 1H; Table S2) (Jaskolka et al., 2021a,b; Smardon et al., 2002). These results suggest that TMEM184B localizes to multiple organellar membranes and prompted us to investigate the hypothesis that TMEM184B is localized broadly within the endosomal system and might play a role in regulating endosomal acidification.

### TMEM184B localizes to early endosomes and late endosomes in human cells

**Fig. 2.**
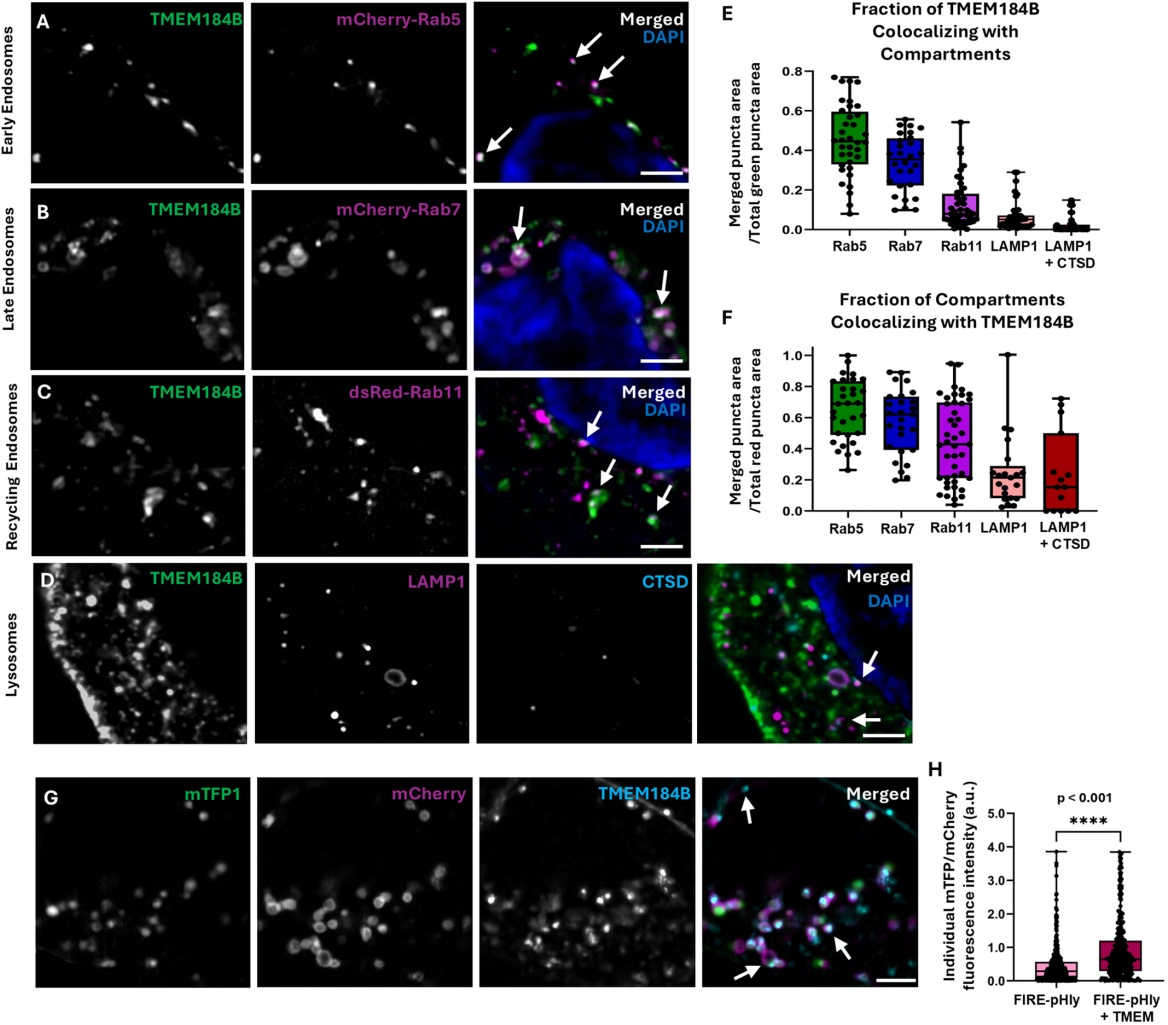
**TMEM184B localizes to early endosomes and late endosomes in human cells.** All images show single slices of HEK293T cells expressing FLAG–TMEM184B–GFP. DAPI (blue) marks the nucleus. For each compartment, three separate cell cultures (biological replicates) were imaged. (A–D) Images depicting localization of FLAG–TMEM184B–GFP to EEs (mCherry–Rab5, *n*=36 cells), LEs (mCherry–Rab7, *n*=38 cells), REs (dsRed–Rab11, *n*=44 cells) and lysosomes (LAMP1+CTSD, *n*=42 cells). White arrows highlight overlapping puncta. Scale bars: 2 µm. (E) Fraction of merged puncta relative to total green TMEM184B puncta area (from A–D). (F) Fraction of merged puncta relative to total red pixel area of compartment markers. (G) Cells expressing FIRE-pHly sensor and TMEM184B. White arrows highlight overlapping puncta (TMEM+). Scale bars: 2 µm. (H) Comparison of mTFP1/mCherry fluorescence intensity in FIRE-pHly^+^TMEM^−^ and FIRE-pHly^+^TMEM^+^ puncta (*n*=22 cells). For box plots, the box represents the 25–75th percentiles, and the median is indicated. The whiskers show the complete range. ****P<0.0001 (unpaired two-tailed Mann–Whitney test). a.u., arbitrary units.

TMEM184B colocalizes with recycling endosomes (REs) in mouse sensory neurons and in fly motor neurons (Bhattacharya et al., 2016; Cho et al., 2022), but its distribution in human cells has not been established. Because many of the TMEM184B interactors we identified reside on endosomal membranes or participate in endosomal trafficking (Fig. 1G,H), we hypothesized that TMEM184B might at least partially localize to these compartments. We first quantified the fraction of TMEM184B^+^ puncta that overlapped with endosomal Rab5a (for early endosome, EEs), Rab7a (for late endosomes, LEs) and Rab11a (REs) relative to the total population of TMEM184B^+^ puncta in each cell. Of all TMEM184B puncta identified, 45% showed EE colocalization, 34% showed LE colocalization and 13% showed RE colocalization (Fig. 2A–E). We further examined the prevalence of TMEM184B on each type of endosome. TMEM184B was present on ∼73% of EEs, ∼59% of LEs and ∼45% of REs. There was a small amount of TMEM184B at the plasma membrane, likely resulting from RE fusion (Fig. 2F).

To accurately assess lysosomal localization, both anti-LAMP1 and -CTSD antibodies were used, as their combined presence identifies mature lysosomes (LAMP1^+^CTSD^+^) versus immature lysosomes or late endosomes (LAMP1^+^) (Fig. 2D) (Cheng et al., 2018; Yap et al., 2022; Yap and Winckler, 2022, 2012) We first quantified the overlap between TMEM184B^+^ and LAMP1^+^CTSD^+^ puncta relative to the total number of TMEM184B^+^ puncta in HEK293T cells. Notably, TMEM184B^+^ puncta exhibited minimal colocalization with LAMP1^+^CTSD^+^ puncta (Fig. 2E). However, 24% of LAMP1^+^ puncta contained TMEM184B, indicating its presence on a subset LEs and/or lysosomes (Fig. 2F). Together, these data indicate that TMEM184B is broadly distributed across endosomal populations, consistent with the IP-MS results.

During the transition from early to late endosomes, a stepwise process occurs in which Rab7a replaces Rab5a on the endosomal membrane (Langemeyer et al., 2020; Mottola, 2014; Skjeldal et al., 2021). Therefore, both Rab GTPases can transiently coexist on the same compartment (van der Beek et al., 2022). This might explain the high fractions of TMEM184B puncta overlapping with Rab5a or Rab7a. In comparison, relatively few TMEM184B puncta were localized to LAMP1^+^ immature or mature lysosomes. These findings indicate that TMEM184B function might be associated with early stages of endocytosis rather than the terminal degradation phase. This could occur if TMEM184B is degraded by lysosomes or retained on LEs during LE–lysosome (LEL) fusion.

We next wanted to determine whether levels of TMEM184B expression in a compartment correlate with their pH. To do this, a pH sensor targeted to LAMP1 (FIRE-pHly) (Chin et al., 2021) was overexpressed in HEK293T cells along with miRFP670-tagged TMEM184B (Fig. 2G). The relative acidity of FIRE-pHly+ compartments with or without overexpression of TMEM184B was measured by the fluorescence intensity ratio of mTFP1 to mCherry. Interestingly, there was a significant increase in the mTFP1-to-mCherry ratio in compartments overexpressing TMEM184B (Fig. 2H), indicating that increased TMEM184B on LAMP1+ compartments reduced acidification. This could occur if TMEM184B overexpression on LAMP1+ compartments disrupted trafficking machinery or V-ATPase subunit delivery. Supporting this idea, endosomal hypo-acidification is also seen in neurological diseases with overexpression of endosomal pumps and transporters, such as the V-ATPase itself (Marshansky and Futai, 2008; Petzoldt et al., 2013), the NHE9 exchanger known as SLC9A9 (Beydoun et al., 2017; Kokane et al., 2025), and proton-activated chloride transporters (Osei-Owusu et al., 2021; Peng et al., 2023). Overexpression of endosomal transporters or channels can mimic either gain-of-function or loss-of-function mutations (Lucien et al., 2017; Xinhan et al., 2011), emphasizing the delicate balance of the electrochemical environment in endosomes and lysosomes.

### TMEM184B mutant neurons show reduced endolysosomal acidification

The putative interactions between TMEM184B and the V-ATPase (Fig. 1D; Tables S1, S2) suggest that TMEM184B might regulate V-ATPase function. The endosomal system comprises a dynamic network of compartments that undergo progressive acidification. To explore this, we utilized FIRE-pHly (Chin et al., 2021), a pH-sensitive sensor tagged to LAMP1, to assess the acidity of both late endosomes and lysosomes (Yap et al., 2022; Yap and Winckler, 2012, 2021, 2022) in primary cortical neurons from *Tmem184b*-mutant and wild-type mouse embryos. Changes in compartment acidification were assessed by the ratio of green fluorescence (mTFP1) to red fluorescence (mCherry) in FIRE-pHly^+^ puncta (G/R ratio).

**Fig. 3.**
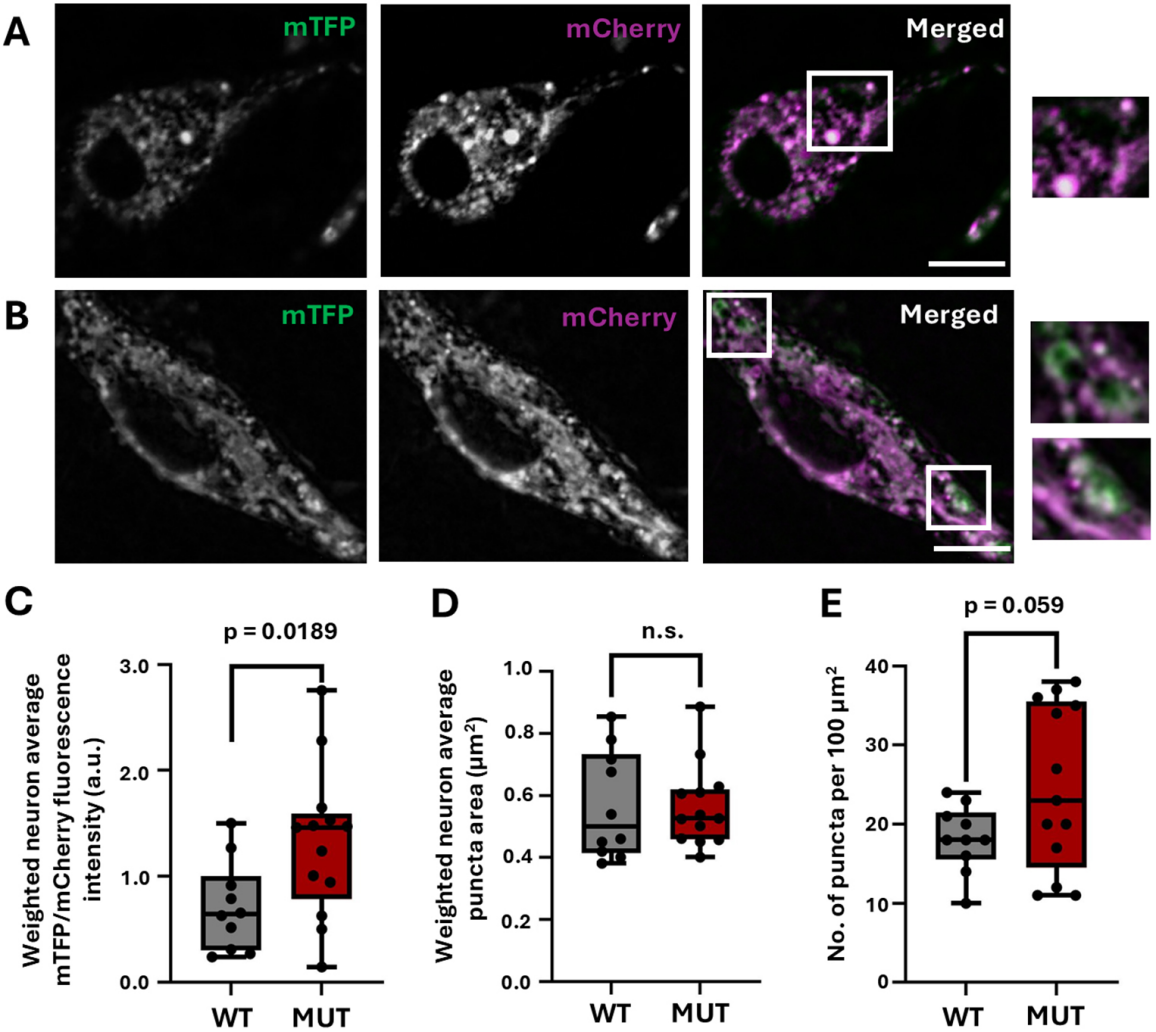
**TMEM184B mutant neurons have reduced endolysosomal acidification.** All images are single slices of the cell body and initial projections. Data from three biological replicates (two or three pooled embryos in each replicate). *N*=10 WT neurons, 13 MUT neurons. (A,B) Comparison of wild-type (A) and *Tmem184b*-mutant (B) cortical neurons transfected with FIRE-pHly. Cropped images show enlarged, abnormal green puncta (white boxes). Scale bars: 5 µm. (C) Comparison of weighted average mTFP1/mCherry fluorescence intensity between wild-type and mutant neurons (*P*=0.0189, unpaired two-tailed *t*-test with Welch's correction). (D) Weighted average puncta area (µm²) for wild-type and mutant neurons [ns, not significant (*P*=0.751), Mann–Whitney test]. (E) Number of puncta normalized to cell body area per 100 µm² for each neuron (*P*=0.059, unpaired *t*-test with Welch's correction). For box plots, the box represents the 25–75th percentiles, and the median is indicated. The whiskers show the complete range. a.u., arbitrary units.

As expected, FIRE-pHly^+^ puncta were primarily localized near the soma (Fig. 3A,B). Notably, we observed that *Tmem184b*-mutant neurons occasionally displayed large puncta with prominent green fluorescence (Fig. 3B). We suspect these abnormal, enlarged compartments represent deacidified LEs or lysosomes, as wild-type neurons did not display similarly shaped puncta. In the absence of TMEM184B, FIRE-pHly^+^ puncta exhibited a significantly increased average G/R fluorescence ratio in each neuron (*P*=0.0189), indicating deacidification of these compartments compared to controls (Fig. 3C). *Tmem184b*-mutant neurons exhibited deacidified puncta regardless of size (Fig. S2). Average puncta size showed no significant difference between *Tmem184b-*mutant neurons and controls (Fig. 3D). Furthermore, no significant change in the density of FIRE-pHly^+^ puncta was observed (Fig. 3E).

Together, these results could suggest a perturbation in LEL fusion, leading to alkaline lysosomes residing in the cell body. An alternative explanation might be that impaired axonal retrograde LE transport reduces LEL fusion in the absence of TMEM184B. An interesting aspect to our findings is that TMEM184B appears to regulate lysosomal acidification without prominent lysosomal localization. It might achieve this by facilitating the budding and trafficking of the V_0_ subcomplex from the Golgi or endosomes, an essential step for V-ATPase assembly on endosomes and lysosomes. Supporting this idea, our IP identified interactors that participate in Golgi transport and vesicular tethering, suggesting that TMEM184B could participate early in the packaging or delivery of V-ATPase subunits from the Golgi throughout the endosomal system. Cellular distribution of lysosomes with respect to the nucleus, which is known to influence their pH, could also be affected (Johnson et al., 2016).

### *Tmem184b*-mutant mice exhibit disrupted assembly of the vesicular proton pump

**Fig. 4.**
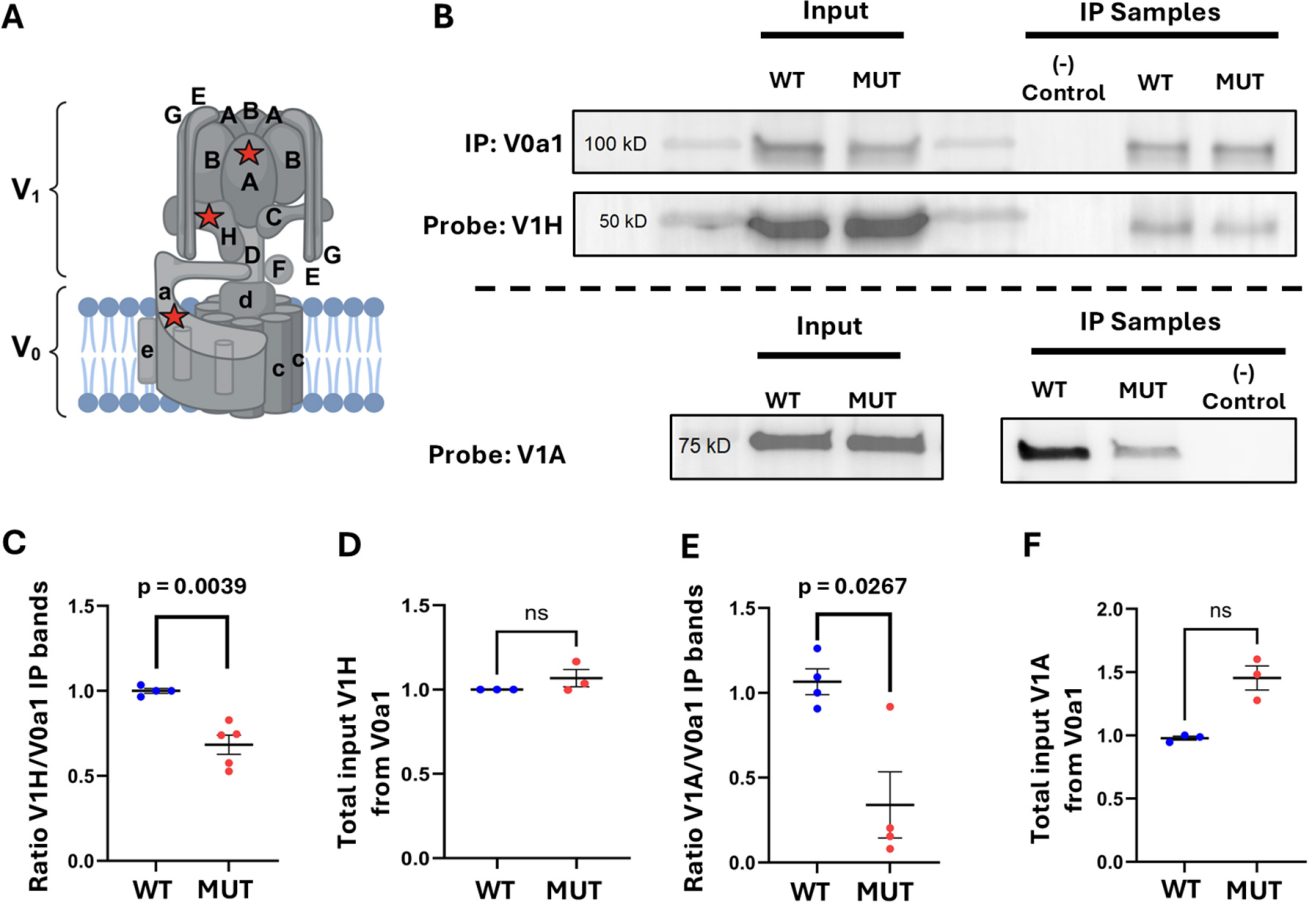
***Tmem184b*-mutants exhibit disrupted assembly of the vesicular proton pump.** (A) Diagram of the vesicular proton pump. Created in BioRender by Wright, E. 2025. https://BioRender.com/qysba04. This figure was sublicensed under CC-BY 4.0 terms. Red stars indicate specific subunits targeted in assembly assay. (B) Representative blots of mouse hippocampal lysates for V1H (top) or V1A (bottom) after V0a1 pulldown in wild-type and mutant mice. (C,E) Quantification of the interaction between V0A1 and V1H (C) or V1A (E) in *Tmem184b-*mutants and wild-type controls. V1 subunit amounts were normalized to the average total of V0A1 band intensity in wild-type IP samples. *P*=0.0039 (C), *P*=0.0028 (E). (D,F) Quantification of total V1H (D) and V1A (F) normalized to the total amount of V0A1 input. *P*=0.32 (D), *P*=0.33 (F). For all graphs, statistical evaluation used unpaired two-tailed *t*-test with Welch's correction; ns, not significant. Error bars represent s.e.m. Dots indicate biological replicates.

V-ATPase activity is regulated by the assembly of its V_0_ and V_1_ subcomplexes. The formation of these complexes is influenced by lumenal nutrient levels and growth signaling pathways, adjusting to the metabolic needs of the cell. As we saw reduced endolysosomal acidification in the absence of TMEM184B, we wanted to determine whether this reduction was due to reduced V-ATPase assembly through co-IP assays. Our results revealed significantly reduced interactions between the subunit V0a1 and both V1A and V1H subunits in *Tmem184b*-mutant mice (Fig. 4A,B) relative to wild-type controls. The interaction between V0a1 and V1H was reduced by 31.72% in the absence of TMEM184B, whereas the interaction between V0a1 and V1A was reduced by 64% (Fig. 4C,E). Quantification of total V1H and V1A levels in lysates indicated that the reduced interaction was not due to a decrease in protein expression (Fig. 4D,F). These findings indicate that the absence of TMEM184B disrupts V-ATPase assembly, which offers a plausible mechanistic explanation for the decreased endolysosomal acidification seen in *Tmem184b*-mutant neurons.

This study identifies a novel role for TMEM184B as a regulator of endolysosomal acidification. However, several aspects of its function remain unclear, including its precise biological role. The classification of TMEM184B within the transporter-opsin-G protein-coupled receptor (TOG) superfamily suggests a potential function as a transporter (Yee et al., 2013). We suspect that the functional role of TMEM184B might affect both localization to the appropriate intracellular compartments and proton pump activity of the V-ATPase, perhaps through separate and possibly indirect mechanisms. Although de-orphanizing putative transporters is technically challenging, identifying the molecular substrates that might be transported by TMEM184B is of significant interest. Some possibilities consistent with our results are that TMEM184B could participate as an endosomal nutrient sensor for the V-ATPase or that it could indirectly modulate V-ATPase activity via ion counterbalance within endosomes. Such investigations are expected to refine the working model of the functional role of TMEM184B and would build upon our results to better understand and treat TMEM184B-associated neurodevelopmental disorders and other nervous system disorders featuring endolysosomal dysfunction.

## MATERIALS AND METHODS

### Experimental design

For human cell TMEM184B localization, ∼15–20 cells were imaged for each experimental group (TMEM184B–FLAG versus GFP–FLAG) across three separate imaging sessions. For endolysosomal acidification assessment, a total of 13 mutant neurons and 10 wild-type primary cortical neurons were analyzed across three separate embryonic neuron dissections. Embryonic neurons were obtained from female mice at embryonic day 16. For V-ATPase assembly evaluation, five *Tmem184b*-mutant (three male, two female) and five wild-type (three male, two female) were euthanized for hippocampus dissection.

### Animals

The use of mice for this study was approved by the University of Arizona IACUC (protocol 17-216). *Tmem184b*-mutant mice used in this study contain a gene-trap insertion originally created by the Texas A&M Institute for Genomic Medicine allele Tmem184bGt^(IST10294F4)^ on the C57BL/6 background. Mice with this insertion exhibit <5% of wild-type TMEM184B mRNA expression by both qPCR and RNAseq (Bhattacharya et al., 2016; Larsen et al., 2022). We therefore call this line a ‘mutant’ rather than a ‘knockout’ throughout the manuscript. C57BL/6 mice (littermates when possible) were used as wild-type controls throughout this study. All mouse husbandry was in accordance with guidelines of the Institutional Animal Care and Use Committee (protocol 17-216) at the University of Arizona.

### Reagents

Antibodies include: mouse monoclonal anti-V5-epitope antibody (Thermo Fisher Scientific, cat. no. R960-25, RRID:AB_2556564, 1:1000), anti-Myc tag (9B11) mouse mAb (Cell Signaling Technology, cat. no. 2276, RRID:AB_331783, 1:1000), rabbit polyclonal anti-ATP6V0A1 (Novus, cat. no. NBP1-89342, RRID:AB_11015740, 1:1000), rabbit polyclonal anti-ATP6V1A (Proteintech cat. no. 17115-1-AP, RRID:AB_2290195, 1:500), rabbit polyclonal anti-ATP6V1H (abcam, #187706, 1:500), rabbit polyclonal anti-ATP6V0D1 (Proteintech, #18274-1-AP), anti-rat-IgG horseradish peroxidase (HRP)-conjugated antibody (R&D Systems, cat. no. HAF005, RRID:AB_1512258, 1:5000), anti-rabbit-IgG HRP-linked antibody (Cell Signaling Technology, cat. no. 7074, RRID:AB_2099233, 1:5000), monoclonal rat anti-LAMP1 (DSHB cat. no. 1d4b, RRID:AB_2134500, 1:500), rabbit monoclonal anti-cathepsin D (Abcam cat. no. ab75852, RRID:AB_1523267, 1:500), Cy3-conjugated goat anti-rabbit IgG (Jackson ImmunoResearch Labs cat. no. 111-165-144, RRID:AB_2338006, 1:500), Cy5-conjugated goat anti-rat IgG (Jackson ImmunoResearch Labs cat. no. 112-175-143, RRID:AB_2338263, 1:500), and purified anti-DYKDDDDK Tag antibody (BioLegend cat. no. 637301, RRID:AB_1134266, 1:500).

For control immunoprecipitations, we used Addgene plasmids pcDNA4/TO-myc-GFP-Flag (Addgene plasmid #82471, deposited by Daniel Schoenberg) and cloned the insert from pEF1a-BirA-V5 (Addgene plasmid #100548, deposited by Jian Xu) into the FCIV backbone, which was a gift from Dr Aaron DiAntonio (Washington University, St. Louis, MO, USA). pcDNA3.1 TMEM184B vectors were ordered from GenScript. FLAG-hTMEM184B-GFP was generated by Twist Biosciences. FLAG-GFP was made in house. mCherry-Rab7a-7 was Addgene plasmid #55127; RRID: Addgene_55127 (deposited by Michael Davidson). mCherry-Rab5a-7 was Addgene plasmid #55126; RRID: Addgene_55126 (deposited by Michael Davidson). DsRed-rab11 WT was Addgene plasmid #12679; RRID: Addgene_12679a (deposited by Richard Pagano). pLJM1-FIRE-pHLy was Addgene plasmid #170775; RRID: Addgene_170775 (deposited by Aimee Kao).

### Cell culture and lysis

HEK293T cells were cultured in high glucose Dulbecco's modified Eagle's medium (DMEM; Gibco) containing 10% fetal bovine serum (Atlas Biologicals #FS-0050-AD), 110 mg/ml sodium pyruvate (Sigma Aldrich) and 10,000 U/ml penicillin-streptomycin (Gibco). Cells were incubated at 5% CO_2_ at 37°C.

For immunoprecipitation coupled with tandem mass spectrometry (IP-MS) analysis and follow-up experiments, cells were removed from incubation and placed on ice for the entire lysis protocol. Medium was aspirated from the plate and replaced twice with ice-cold PBS (Gibco) to gently wash cells. Cell scrapers (RPI) were used to pool cells for transfer to clean 1.5 ml Eppendorf tubes. Cells were gently centrifuged at 800 ***g*** for 2 min. Cell pellets were resuspended with RIPA lysis buffer (150 mM NaCl, 5 mM EDTA, 50 mM Tris, 1.0% Triton X-100, 0.5% sodium deoxycholate, 0.1% SDS) containing EDTA-free protease inhibitor (Biomake). Cells were incubated at 4°C for 1 h on a rotator followed by centrifugation at 21,000 ***g*** for 5 min. Supernatants were collected and passed through a 29-gauge syringe (Exel International, 26028) seven times on ice prior to protein content quantification using a Pierce BCA protein assay kit (Thermo Fisher Scientific). Cells were stored at −80°C for future IP-MS experiments.

### Primary cortical neuron culture

Cortices were collected from *Tmem184b-*mutant and wild-type mice at embryonic day 16 from dams (see below for dissection details). Neurons were dissociated with 0.25% trypsin (Gibco) for 15 min at 37°C. Trypsin was replaced with 10% fetal bovine serum (FBS)-containing medium and incubated for 3 min at room temperature. Neurons were washed three times with ice-cold HBSS (Gibco) and triturated with fire polished glass Pasteur pipets (Thermo Fisher Scientific) of decreasing diameter 10 times each. Suspended neurons were filtered through 40 µm cell strainers (Chemglass) and resuspended in Neurobasal Medium (Gibco) containing 0.5 mM L-glutamine, 10,000 U/ml penicillin-streptomycin (Gibco) and 1× B27 supplement (Gibco). Approximately 20,000 neurons were plated in eight-well chambered #1.5 cover glass (Thermo Fisher Scientific) previously coated with 0.15 μg/ml poly-D-lysine. Half of the medium was replaced the next day prior to transfections. Neurons were incubated at 5% CO_2_ at 37°C.

### Transfections

Transfections in HEK293T cells were done using GeneJuice (EMD Millipore) and serum-free OptiMem GLUTAMAX (Gibco). Expression vectors of TMEM184B variants were tagged with either the c-Myc epitope tag or the V5 epitope tag sequence. Control vectors included bifunctional ligase/repressor (BirA) tagged with the Myc tag or V5 tag. Each vector was then transfected into in duplicate.

To assess TMEM184B localization in the endosomal system, HEK293T cells were transfected with pHIV-FLAG-TMEM184B-myc or FLAG-GFP. To mark endosomes, cells were transfected with mCherry–Rab7 (LEs), mCherry–Rab5 (EEs) or dsRed–Rab11 (REs) (Choudhury et al., 2002). Lysosome markers (CTSD and LAMP1) were evaluated using immunocytochemistry.

Transfections in primary cortical neurons were done using FuGENE HD transfection reagent (Promega) and OptiMem. After transfection with pFUGW-FIRE-pHly for pH analysis, neurons were incubated for 6 h before the medium was changed. Imaging was performed 2 days after transfections.

### Immunocytochemistry

HEK293T cells were gently washed three times with 1× PBS-1% Tween 20 (PBST). Cells were fixed with 4% paraformaldehyde (PFA) in PBS for 15 min on ice. PFA was removed, cells were washed with 1× PBST for 5 min twice. Blocking solution [5% normal goat serum (Fisher Scientific) in 1× PBST] was applied to cells and incubated for 30 min at room temperature on a rotator. Cells were gently washed three times with 1× PBST for 5 min to remove the remaining blocking solution. Primary antibodies against LAMP1 and Cathepsin D in 0.3% NGS and 1× PBST were applied to cells. Chambered slides were wrapped in parafilm and incubated overnight at 4°C. Primary antibodies were removed, and cells were gently washed three times with 1× PBST for 5 min. Secondary antibodies, goat anti-rat-IgG Cy5 and anti-rabbit-IgG Cy3 in 1× PBST, were added to cells. Chambered slides were covered in foil and incubated at room temperature for 1 h. Cells were gently washed three times with 1× PBST for 5 min to remove any remaining secondary antibody and once with 1X PBS. Vectashield+DAPI (Fisher Scientific) was added to slides prior to applying #1.5 micro cover glass (Electron Microscopy Sciences)

### Hippocampus dissection

Mice were humanely euthanized with carbon dioxide, and hippocampi were removed within 30 min of euthanasia. Isolated hippocampi were immediately placed into RIPA lysis buffer with protease inhibitor. Hippocampi were crushed using clean pestles and sonicated before centrifugation at 21,000 ***g*** for 5 min. Protein concentrations from the final supernatant were quantified using a Pierce BCA protein assay kit. Hippocampi were collected from total of eight *Tmem184b*-mutant (five male, three female) and eight wild-type controls (five male, three female). All mice were ∼6 months old.

### Immunoprecipitation

Constructs containing mouse TMEM184B (NP_766196.1, 407 amino acids) or human TMEM184B (NP_036396.2, 407 amino acids) were used as appropriate. Mouse and human TMEM184B sequences are 96% identical (391/407) and 97% similar (395/407). 500 μg of Protein A/G Magnetic Beads (MedChemExpress, HY-K0202) were loaded into 1.5 ml Eppendorf tubes and washed three times with PBS prior to use. Beads were conjugated with antibody and incubated for 2 h at room temperature on a rotator. Bead–antibody conjugates were washed four times with 1× PBS before addition of lysates and incubated overnight at 4°C on a rotator. Lysate supernatants were removed, and antibody–antigen conjugates were washed four times with PBS and transferred to new 1.5 ml Eppendorf tubes to avoid non-specific binding to any remaining lysates. Proteins were eluded twice with 1× Laemmli sample buffer (Cold Spring Harbor) for a final volume of 40 μl. Eluents were incubated for 5 min (95°C or 37°C for TMEM184B samples) prior to loading into gel.

For mass spectrometry analyses, 5 µg of anti-V5-epitope antibody (Invitrogen) was used for bead conjugation. 1.5 mg whole-cell lysates were added to conjugated beads prior to incubation overnight. For V-ATPase assembly analysis, 0.16 µg anti-ATP6V0A1 and 0.20 µg anti-ATP6V0D1 primary antibodies were used to conjugate beads. 100 µg of hippocampus protein was added to conjugated beads.

### Gel electrophoresis, Coomassie staining and immunoblotting

For V-ATPase assembly analysis, whole hippocampus lysates were mixed with 1× Laemmli sample buffer and incubated for 5 min at 95°C. Whole lysate controls and IP eluents were loaded into 4–20% Mini-PROTEAN TGX protein gels (Bio-Rad). Proteins were transferred onto 0.45 m PVDF transfer membranes (Thermo Fisher Scientific) and blocked with 5% casein in 1× TBST (Sigma-Aldrich). Membranes were incubated overnight at 4°C in primary antibody against ATP6V1H (1:500) or ATP6V1A (1:500) in 0.3% casein and 1× TBST. Primary antibodies were removed, and membranes washed before incubation with HRP-conjugated antibody. Blots were developed using ECL blotting substrate (Bio-Rad) and quantified using Image Lab software (Bio-Rad). For IP samples, V1A and V1H quantification was normalized to the total amount of V0A1 in the IP lane. Whole-cell lysate controls were normalized to total amount of protein loaded into the sample lane. Blot transparency data are shown in Fig. S3.

For IP-MS, eluents were run fresh on the day of MS analysis. IP-MS gels were stained with mass-spectrometry grade Coomassie Blue, imaged for annotation purposes, and processed and analyzed by the University of Arizona College of Medicine's Quantitative Proteomics Laboratory.

### Mass spectrometry data acquisition and analysis

To identify true TMEM184B protein–protein interaction candidates, a multiple epitope tags co-IP approach was used, whereby two separate plasmids were created containing the same TMEM184B protein coding sequence but with either a Myc tag sequence or a V5 tag sequence at the end of the TMEM184B C-terminus. In parallel, HEK293T 100 mm plates were transfected with one vector in duplicate; thus, eight plates in total were transfected: duplicates of TMEM184B–Myc, TMEM184B–V5, GFP–Myc and BirA–V5 equating to two biological replicates per sample. Mass spectrometry data was acquired as previously described (Kruse et al., 2017; Parker et al., 2019). Once acquired, Scaffold software-derived (version 5, Proteome Software Inc., Portland, OR) total spectrum counts (TSCs) were compiled into a CSV and uploaded into the RStudio GUI (version 4.1.0) using the 64bit R software (version 4.0.4). Mathematical operations on TSCs were performed in the programming language R (Choi et al., 2011).

To analyze the resulting MS data, we filtered to remove low abundance proteins (<5 TSCs in both TMEM184B IP duplicates and <10 TSCs in every TMEM184B sample) (Teo et al., 2013)). Following filtering, subsequent processing was performed on each tag dataset separately. Values of 0.1 were added to proteins with 0 TSCs to enable fold change calculation (Choi et al., 2011; Sardiu et al., 2008; Sowa et al., 2009). TSCs were then normalized by molecular weight (MW) and averaged between duplicates. These averages were used to calculate fold changes (FCs), one for each tag (e.g. average TMEM184B–Myc MW-normalized TSC/average GFP–Myc MW-normalized TSC), and a combined FC (e.g. average of all TMEM184B MW-normalized TSC/average of all control bait MW-normalized TSC). Candidates with ≥2 average FC in both tag system and a ≥2 combined FC were retained, and common contaminants were filtered out as previously described (Mellacheruvu et al., 2013). R code used for this analysis is available on Github (https://github.com/eriklarsen4/Endo).

### Fluorescence microscopy

All imaging was taken on the Nikon Spinning-Disk SoRa super-resolution microscope in the University of Arizona Cancer Center (UACC) Microscopy Shared Resource. Images were acquired on a Nikon CSU-W1 SoRa Spinning-Disk Confocal microscope equipped with a Photometrics Kinetix sCMOS camera. Single slice images were acquired using a Nikon 60× Plan Apo 1.40 NA objective lens.

Prior to imaging live primary neurons, neurobasal medium was removed from eight-well cover glass chambers and replaced with pre-warmed Live Cell Imaging Solution (Thermo Fisher Scientific). Neurons were allowed to acclimate to the microscope live-cell imaging chamber (5% CO_2_ and 37°C) for 30 min prior to imaging. The 488 and 561 nm lasers were used to excite mTFP1 and mCherry, respectively. Single slices were obtained and exported as ND2 files for later analysis in Nikon NIS Elements AR. Wild-type (10) and *Tmem184b*-mutant (13) neurons were imaged across three separate groups of one or two pooled embryos per genotype.

For HEK293T localization experiments, the 405, 488 and 640 nm lasers were used to excite DAPI, GFP and Cy5, respectively. The 561 nm laser was used to excite mCherry, dsRed or Cy3 according to the specific compartment. Single-slice images were acquired for analysis in ImageJ (FIJI). For each compartment assessed, 15–20 images were taken for TMEM184B and GFP controls each.

### Colocalization analysis

Raw CZI files were imported into ImageJ (FIJI) for processing, including background subtraction, prior to analysis. To assess the colocalization of TMEM184B with other cellular compartments, images from the red and green channels were binarized and segmented using the watershed function. Overlapping puncta between the binarized images were identified and analyzed using the ‘AND’ function in the Image Calculator. Both the merged puncta and individual puncta from each channel were quantified using the ‘Analyze Particles’ function to determine puncta area (µm²). The degree of TMEM184B localization to each compartment was calculated by dividing the area of merged puncta by the total area of TMEM184B+ puncta. For lysosome localization, images labeled with cathepsin D (CTSD) and lysosomal-associated membrane protein 1 (LAMP1) were first processed using the ‘AND’ function to identify puncta associated with lysosomes. This resulting image was then intersected with TMEM184B+ puncta to assess colocalization. Additionally, separate calculations for LAMP1 and TMEM184B were performed to examine TMEM184B localization to late endosomes or deacidified lysosomes, distinguishing these from conventional lysosomal compartments.

### FIRE-pHly puncta analysis

Nikon NIS Elements AR 5.42.03 software with the General Analysis 3 (GA3) module was used for image processing and analysis. To quantify red channel detections within each cell, a new image channel was created by applying a gaussian filter (sigma=8) and rolling ball background subtraction (radius=18 µm) using the green channel. A signal threshold was applied to this ‘Cell Body’ channel to properly segment the cell body and only quantify the red channel detections within each cell. The following image preprocessing tools were used for both the green and red channels: low pass filter=4 px and rolling ball background subtraction radius=1.2 µm. The Bright Spots Detection tool (diameter=1.5 µm) was used to detect the red channel within the Cell Body and the intensity threshold was adjusted per image to achieve accurate segmentation. The following data was quantified for each cell: cell body area (µm^2^), total channel detections and mean intensity of green and red fluorescence. Weights were determined as follows: dividing the number of puncta within each cell by all the puncta in the study and then multiplying the fraction within each cell by 10 to re-scale the value of the weight to near that for a whole mouse. The green-to-red (G/R) fluorescence ratio for each identified puncta was calculated by dividing the mean green fluorescence intensity by the mean red fluorescence intensity.

### Statistical analysis

All statistical analyses were performed in GraphPad Prism, and *P*-values less than 0.05 were considered statistically significant. Statistical significance was defined as follows: *P*≤0.05 (*), *P*≤0.01 (**), *P*≤0.001 (***). Data are presented as the mean±s.e.m. For FIRE-pHly analysis, significance between wild-type and *Tmem184b-*mutant average puncta size was assessed using a Mann–Whitney test. All other analyses comparing *Tmem184b*-mutant and wild-type samples were evaluated by unpaired two-tailed *t*-tests with Welch's correction.

## Supplementary Material

10.1242/joces.263908_sup1 Supplementary information

Table S1.

Table S2.
