# Supplementary material for: TMEM184B modulates endolysosomal acidification via the vesicular proton pump
Source: J Cell Sci. 2025 Aug 8;138(15):jcs263908. doi: 10.1242/jcs.263908 (PMC12377713; doi:10.1242/jcs.263908)
Supplement: Supplementary information [file joces-138-263908-s1.pdf]

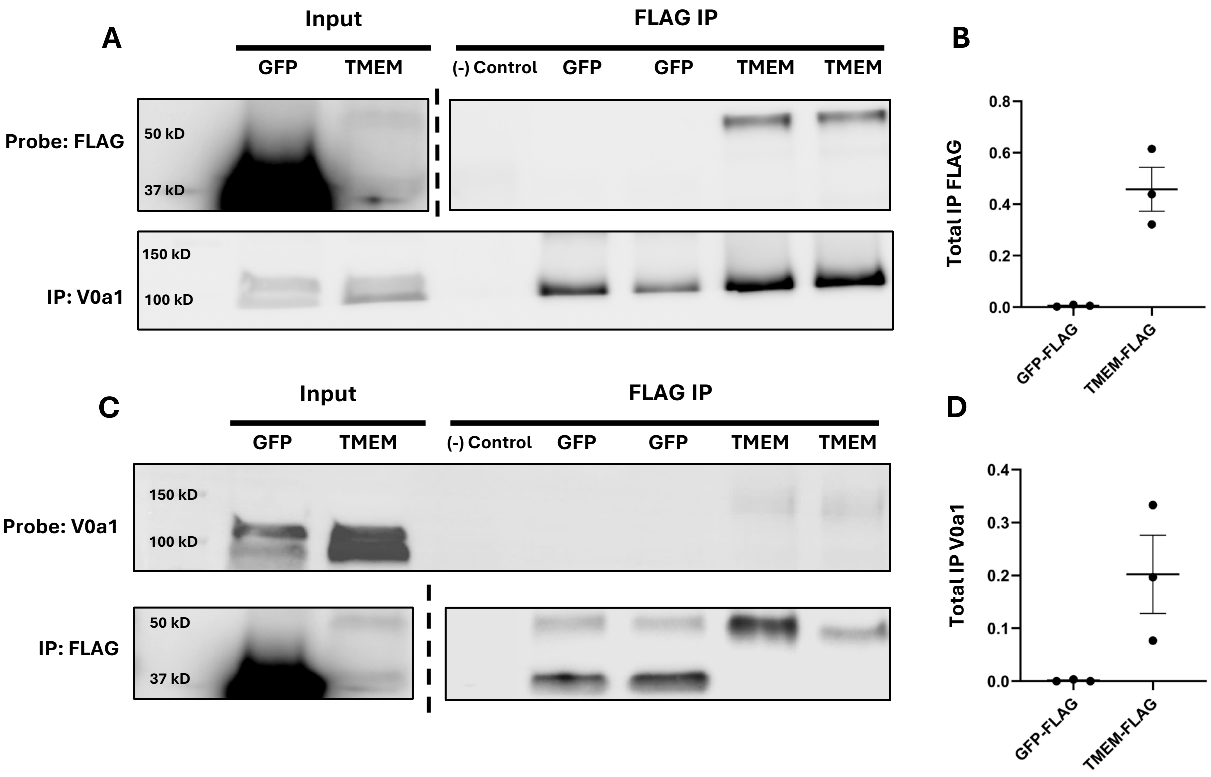

Fig. S1. Validation of interactions between V-ATPase subunit V0A1 and TMEM184B.

(A) Representative blots of FLAG-tagged protein content following V0a1 subunit pulldown in FLAG-GFP and FLAG-TMEM184B expressing cells (n = 3 biological replicates per group). **(B)** Quantification of FLAG signal, normalized to total V0a1 protein in corresponding lane. Error bars represent SEM. **(C)** Representative blots showing V0a1 content following FLAG pulldown in FLAG-TMEM184B and FLAG-GFP expressing cells (n = 3 biological replicates per group). **(D)** Quantification of V0a1 bands, normalized to total FLAG-tagged protein in corresponding lane.

Journal of Cell Science • Supplementary information

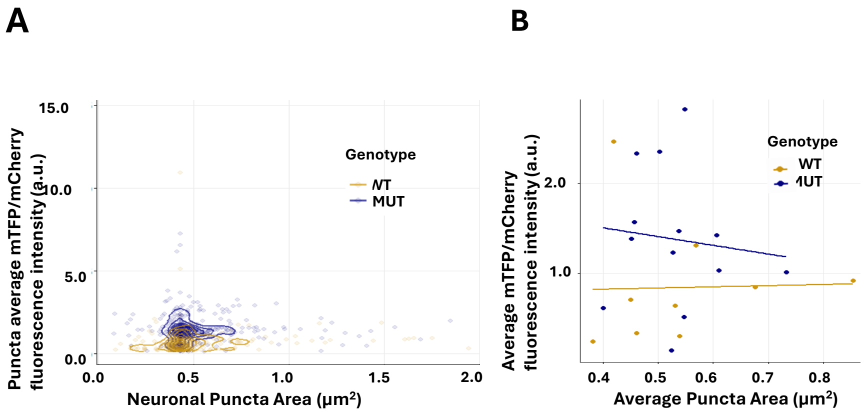

Fig. S2. Comparison of puncta characteristics between wild-type and *Tmem184b*-mutant neurons.

Yellow dots represent wild-type neurons, and blue dots represent*Tmem184b*-mutant neurons. **(A)** Distribution of individual puncta area (µm²) versus corresponding G/R fluorescence intensity. **(B)** Weighted average puncta area per neuron versus average G/R fluorescence ratio.

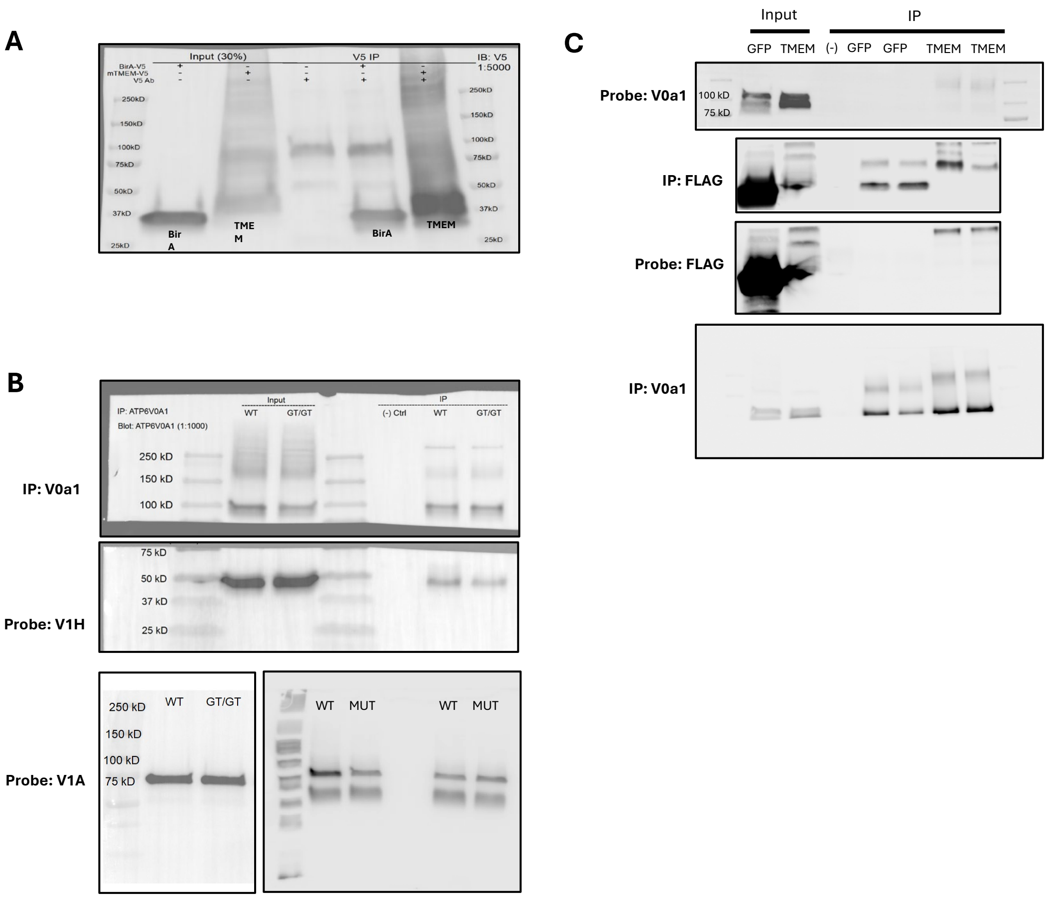

Fig. S3. Blot transparency.

(A) Representative blots from Fig. 1 showing control or TMEM184B content following V5 pulldown in V5-TMEM184B and V5-BirA expressing cells **(B)** Representative blots from Fig. 4 showing V0a1, V1H, and V1A content following V0a1 pulldown in wild-type and *Tmem184b*-mutant mice **(C)** Representative blots from Fig. S1 showing V0a1 content following FLAG pulldown (top) and FLAG content following V0a1 pulldown (bottom).

Journal of Cell Science • Supplementary information

Table S1.

Available for download at
https://journals.biologists.com/jcs/article-lookup/doi/10.1242/jcs.263908#supplementary-data

Table S2.

Available for download at
https://journals.biologists.com/jcs/article-lookup/doi/10.1242/jcs.263908#supplementary-data
